# Reevaluation of Neoadjuvant Chemotherapy for Esophageal Squamous Cell Carcinoma: A Meta-Analysis of Randomized Controlled Trials Over the Past 20 Years

**DOI:** 10.1097/MD.0000000000001102

**Published:** 2015-07-13

**Authors:** Yan Zheng, Yin Li, Xianben Liu, Haibo Sun, Zongfei Wang, Ruixiang Zhang

**Affiliations:** From the Department of Thoracic Surgery, The Affiliated Cancer Hospital of Zhengzhou University, Henan Cancer Hospital, Zhengzhou, Henan, P. R. China.

## Abstract

The effect of neoadjuvant chemotherapy on the survival of patients with thoracic esophageal squamous cell carcinomas (ESCCs) remains controversial. The optimal management strategy for resectable ESCCs varies regionally based on local randomized controlled trials. A systematic review and meta-analysis was conducted to re-evaluate this controversial issue.

A systematic review of the Medline, Embase, and PubMed databases was carried out on data collected between August 1994 and August 2014 to evaluate the role of neoadjuvant chemotherapy. Only randomized controlled trials comparing the effects of neoadjuvant chemotherapy with that of surgery and surgery plus adjuvant chemotherapy were selected.

Six studies with a total of 1202 patients were identified, consisting of a neoadjuvant chemotherapy arm (n = 597) and a surgery alone and surgery plus adjuvant chemotherapy arm (n = 605). The 5-year overall survival benefit for neoadjuvant chemotherapy was statistically significant at α = 0.1 (hazard ratio = 0.81, 95% confidence intervals, 0.65–1.00, *P* = 0.053). All 6 trials recruited patients for more than 5 years with undefined lymphadenectomies. Cisplatin and fluorouracil were adopted as neoadjuvant chemotherapy regimens.

The role of neoadjuvant chemotherapy for ESCC is worth re-investigating. The design of randomized controlled trials should adopt new chemotherapy regimens as well as define the surgical procedure and the details of the lymphadenectomy.

## INTRODUCTION

Evidence from meta-analyses and randomized controlled trials (RCT) supports the survival benefits of neoadjuvant chemoradiotherapy (NACR) for esophageal squamous cell carcinoma (ESCC).^1–4^ However, accumulating evidence suggests a significant level of toxicity results from chemoradiotherapy for ESCC. Specifically, NACR resulted in significant total postoperative mortality (hazard ratio [HR] = 1.95, 95% confidence intervals [CI] = 1.06–3.60, *P* = 0.032),^5^ treatment-related mortality (HR = 1.97, 95% CI = 1.07–3.64, *P* = 0.030),^5^ and postoperative mortality (11.1% versus 3.4%, *P* = 0.049).^6^ The other neoadjuvant therapeutic strategy that has been demonstrated by many studies to be safe for ESCC is neoadjuvant chemotherapy (NAC).^3,5^ There have been several well-designed RCTs and meta-analyses in the Western world; however, the survival benefit of NAC remains controversial. Two multicenter trials^7,8^ and 2 meta-analyses^2,3^ revealed no additive benefit on overall survival (OS) when using NAC for ESCC. Therefore, neoadjuvant and definitive chemoradiotherapy followed by surgery is the standard treatment in Western countries. However, based on the results of local RCTs (level A evidence), the standard management for resectable ESCC in Japan is NAC.^9,10^ There is no general consensus on the role of NAC in ESCC worldwide. The 2 largest trials from western countries showed contradictory outcomes^7,8^ that are difficult to explain. How should the best neoadjuvant method for ESCC be chosen based on contradictory level A evidence? This systemic review will focus on the details of the chemotherapy regimens and surgical procedures from 6 RCTs of operable ESSC over the past 20 years. We aim to elucidate the effectiveness of NAC on survival in ESCC and attempt to explain the contradictory results obtained from different RCTs.

## METHODS

### Ethics Statement

This study was approved by the Research Ethics Committee of the Affiliated Cancer Hospital of Zhengzhou University/Henan Cancer Hospital.

### Search Strategy

Medline (August 1994–August 2014), Embase (August 1994–August 2014), and PubMed (August 1994–August 2014) databases were systematically queried for literature by 2 independent reviewers. “Esophageal neoplasms” [Medical subject heading (MeSH)] was combined with “chemotherapy, neoadjuvant” (MeSH), and “preoperative,” “neoadjuvant,” and “chemotherapy” were used as text words.

### Inclusion and Exclusion Criteria

Articles were included if they were RCTs comparing surgery plus NAC with surgery alone and surgery plus adjuvant chemotherapy in patients with resectable thoracic ESCC. Abstracts and fully published reports with data on survival were included. The publication language was limited to English. Reports on cervical esophagus carcinomas were excluded. Two reviewers performed the methodological quality assessment independently, and a third reviewer was employed when there were disagreements between the reviewers.

### Data Analysis

STATA version 12 (StataCorp, College Station, TX) was used to perform meta-analyses. The statistical heterogeneity for each pooled estimate was quantified and assessed by Cochran's *χ*^2^ statistic and the *I*^2^ statistic, respectively. If heterogeneity existed, a random effects model was used; otherwise, a fixed effects model was employed. STATA version 12 was used to perform the pool analysis. The Mantel–Haenszel model was used and reported as HR with 95% CIs to assess the influence of NAC on OS. The significance of the pooled HR was determined by the *Z*-test. *P* < 0.05 was considered to be statistically significant. If possible, the HR and associated variances were obtained directly from each article. Unreported HRs were calculated by extraction of summary statistics from the Kaplan–Meier curve according to methods by Parmer et al^11^ and Tierney et al.^12^ There was no Kaplan–Meier curve of ESCC in Kelsen reports.^7,13^ We used the HR and 95% CI reported by Sjoquist^3^ for the ESCC subgroup in the 8911 trial. The potential publication bias was assessed by the Begg's test and Egger's test by using STATA version 12.

## RESULTS

**FIGURE 1:**
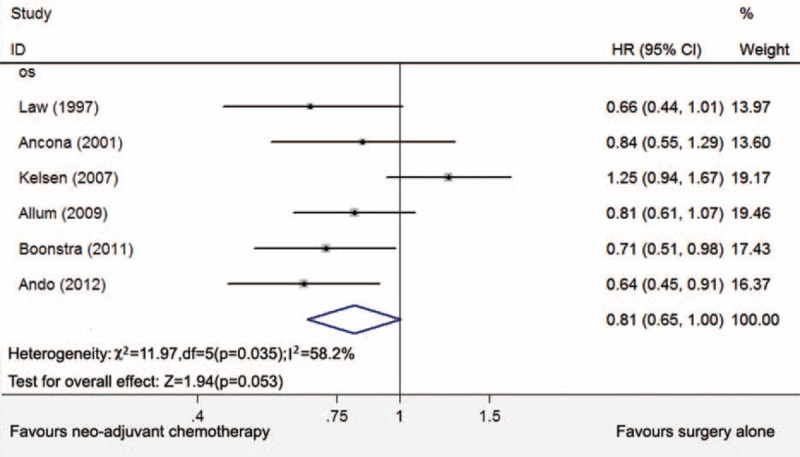
Five-year overall survival for NAC with surgery or surgery with adjuvant chemotherapy. NAC = neoadjuvant chemotherapy.

Six studies that were randomized comparisons of NAC versus surgery and surgery plus adjuvant chemotherapy (n = 1202) were included. The main characteristics and resection rates of eligible studies are shown in Table 1. Squamous cell carcinoma was selected as the histopathology for the entire population. Two studies enrolled patients with adenocarcinoma (66.5%^8^ and 53.3%^7^); however, we only selected the ESCC subgroup from these studies. The HR for the comparison of NAC with surgery for the treatment of ESCC was used to access the treatment effects. As Figure 1 shows, there was no statistically significant benefit for NAC in a pooled analysis at *α* = 0.05 (HR = 0.81, 95% CI = 0.65–1.00, *P* = 0.053); however, NAC was significantly beneficial at *α* = 0.1. Begg's and Egger's tests showed no publication bias for the combined analysis (Begg's test, *P* = 0.707; Egger's test, *P* = 0.307). The NAC regimens and surgical procedures are summarized in Tables 2 and 3, respectively. The interval between first cycle of NAC and surgery was approximately 8 weeks. The enrolment periods were 5 to 7 years. The lymphadenectomy procedure was the most variable part of the operation among the eligible studies, and the lymphadenectomy strategy was not well described.

**TABLE 1:**
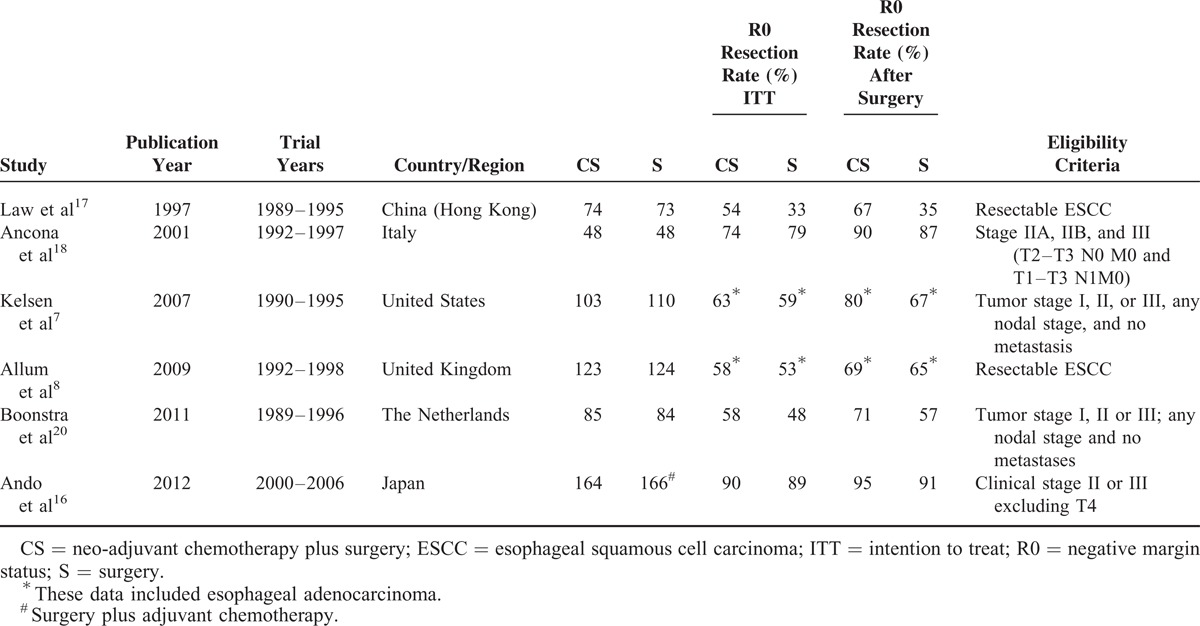
General Details and Resection Rates of 6 Eligible Studies

**TABLE 2:**
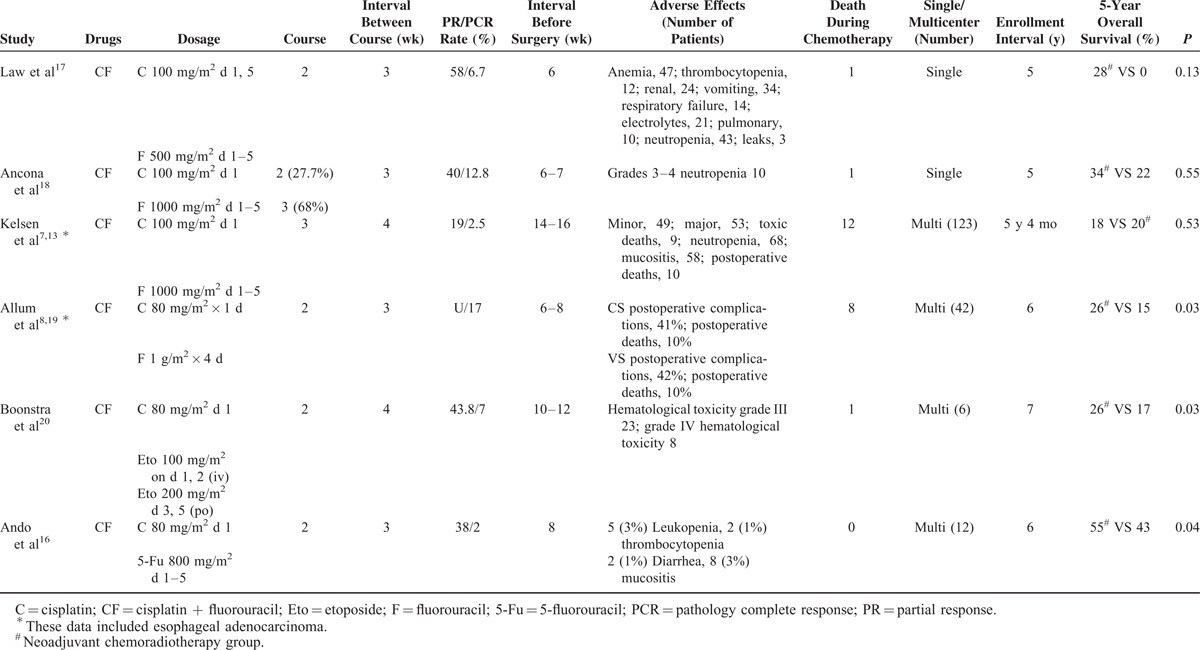
Neoadjuvant Chemotherapy Regimens in 6 Randomized Trials Included in the Meta-Analysis

**TABLE 3:**
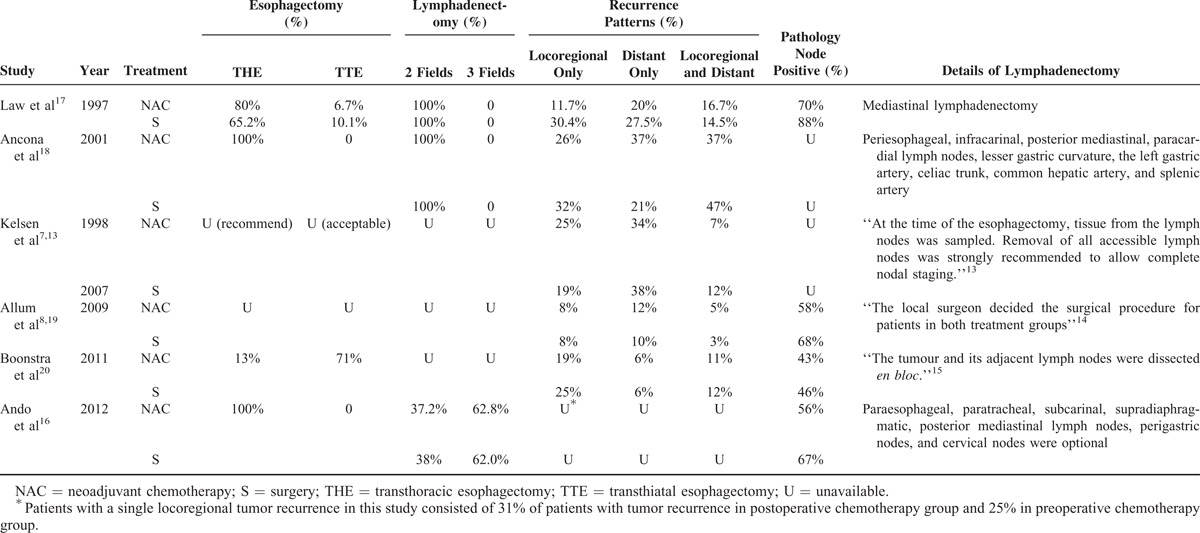
Comparison of Different Surgical Procedure of NAC and S Group in 6 Eligible Studies

## DISCUSSION

The OS of patients with resectable esophageal carcinoma remains poor, with a 5-year survival of 15% to 34%,^8^ depending on the region. Multimodal treatments for resectable esophageal carcinoma have been explored. Patients with esophageal carcinoma often have a poor postoperative performance status due to the reconstruction of digestive ducts. Generally, they tolerate preoperative (neoadjuvant) therapy much better than postoperative (adjuvant) therapy.^14^ Therefore, neoadjuvant therapy has been extensively studied with RCTs. However, compared with other solid tumors, it seems more difficult for esophageal carcinoma to have consent worldwide. Different countries and regions have different therapeutic strategies based on the results of local RCTs. It has been demonstrated that NACR could confer survival benefits over surgery alone by several clinical trials^1,4^ and meta-analyses,^7,8^ and it serves as a standard treatment in western countries. However, the associated toxicity of NACR is a problem for ESCC. Kumagai et al suggested a significantly higher risk of total postoperative mortality and treatment-related mortality for ESCC after NACR.^5^ The clinical trial 9901 conducted by Francophone de Cancérologie Digestive (FFCD) compared NACR with surgery alone and was 70.3% patients with ESCC. NACR did not offer any survival benefit (HR = 0.99; 95% CI = 0.69–1.40; *P* = .94), but postoperative mortality was significantly increased (11.1% versus 3.4%; *P* = 0.049).^6^ The known risk factors for ESCC are alcohol and tobacco. Some researchers have suggested that these risk factors increase the risk of cardiopulmonary complications after chemoradiotherapy.^15^ NAC is a standard therapy in Japan based on the Japan Clinical Oncology Group trial 9907 (JCOG9907) trial, which revealed significant survival benefits.^16^ Many clinical trials and meta-analysis have concluded that NAC is a safe strategy with a tolerable level of toxicity.^3,5^ Therefore, the effectiveness of NAC on ESCC should be re-evaluated, and its use for the treatment of ESCC should be reconsidered.

Six RCTs with 1202 cases in last 20 years were included in this study.^7,8,13,16–20^ We attempted to evaluate every detail of the chemotherapy regimens and surgical procedures to determine the source of the opposing and controversial results. In comparison to a previous meta-analysis,^3^ we only included the clinical trials published in the past 20 years. Compared with a meta-analysis by Sjoquist,^3^ we discarded the study published by Nygaard and Schlag published in 1992.^21^ The complete resection rates were 44% in the NAC plus surgery group and 37% in the surgery alone group,^21^ compared with 44% and 45% in the study by Schlag et al in 1992.^22^. Over the past 20 years, the complete resection rate has significantly improved. For the NAC strategy, surgery was adopted only for local control. The low complete resection rate might be a confounding factor in the evaluation of the effectiveness of NAC and may dilute the survival benefits.

As shown in Table 1, 3 of the RCTs enrolled small numbers of patients with ESCC (n ≤ 100).^17,18,20^ One was closed due to low recruitment efficiency.^18^ Three of the largest trials had adequate power to detect modest differences in survival^7,8,13,16,19^; the contradictory outcomes were found among these trials. No survival advantage was detected by the North American intergroup trial for ESCC (Radiation Therapy Oncology Group, RTOG Trial 8911 or USA Intergroup 113), reported by Kelsen.^7,13^ The United Kingdom's Medical Research Council (MRC) trial reported a significant survival advantage for NAC for EC. However, subgroup analysis revealed no significant difference for ESCC (*P* = 0.1).^8,19^ These large RCTs were performed in the early 1990s and reflected the methods in clinical practice during that period. Some chemotherapy regimens are no longer employed for the treatment of patients with ESCC.^20^ The JCOG9907 detected a significant survival benefit by NAC compared with postoperative chemotherapy for ESCC.^16^ In Table 2, the interval between chemotherapy and surgery was longer in the 8911 trial, and a lower pathology complete response (PCR) rate was observed.^7,13^ Three cycles of chemotherapy was used in the trial, whereas 2 cycles were employed by the other trials. All 3 chemotherapy cycles were completed by 71% of the patients.^1,13^ Some researchers suggested NAC to patients who did not respond, delaying the surgical treatment and leading to worse survival. A longer interval may be harmful due to delays in the definitive treatment with surgery.^5^ NAC has associated treatment toxicity. In addition, this was the only trial to report grades 3 and 4 neutropenia toxicity in 29% of patients.^1,13^ It also has been suggested that the higher dose of chemotherapy used in the 8911 trial might be another detrimental problem.^10^ The enrolment interval of all trials was more than 5 years. In multicenter trials, some centers may enroll <1 patient in 1 year, which may affect the heterogeneity of the surgical procedure.

Table 3 shows the surgical procedure details reported in the 6 trials. The operative approach, radicality of resection and methods of reconstruction are major controversies in the surgical treatment of esophageal cancer. Of the 3 larger multicenter clinical trials, the 8911 trial did not report the exact numbers or the surgical type, and the MRC trial did not describe the type of surgical resection clearly. One of the most common failures of these trials was local recurrence. As only local control methods exist in the NAC strategy for treating ESCC, too little attention is given to standard the surgical procedure. Different surgical treatments may significantly affect the survival rate and conceal the benefits from chemotherapy. None of the trials reported the details of mediastinal lymphadenectomies. In a retrospective analysis of our center, it was found that the rate of recurrent nerve lymph node metastasis was 22.6% for the right side and 11.6% for the left side. Thus, if lymphadenectomies of recurrent nerve nodes were not included, we could hardly say it was a negative margin status (R0) resection. Another level of local control would be necessary. The rates of local recurrence are shown in Table 3. The JCOG9907 trial detected a significant survival benefit using NAC. The lower rate of locoregional recurrence in the 9907 trial is shown in Table 3 and might be the result of their meticulous surgical procedures. The R0 resection rates of the other 5 trials were much lower. In the MRC trial, survival of the surgery alone group was poor (median, 13 months).^8,19^ Overall, these trials suggest that NAC is a good strategy if surgical treatment can achieve sufficient local tumor control. Otherwise, radiotherapy should be added as an additional local control strategy.

Compared with other solid tumors that have different multidisciplinary methods among different countries, the lymphadenectomy of gastric carcinoma (GC) is well defined. Based on the magic trial, European countries adopted a treatment strategy including D1 lymphadenectomy plus NAC.^23^ From the results of the ACTS-GC^24^ and CLASSIC^25^ trials, Asian countries implemented D2 lymphadenectomy plus adjuvant chemotherapy as the standard therapy for GC. D0/D1 lymphadenectomy with adjuvant chemoradiotherapy is the accepted treatment strategy in America based on the INT 0116 trial.^26^ Different adjuvant therapies were adopted depending on the type of lymphadenectomy. The results of combined therapies cannot be discussed without regard to the surgical procedure employed. Thus, it is easily to understand why the treatment strategy for GC varies in different countries.

### Meta-Analysis of NAC and Survival for Patients With ESCC

All 6 studies were included to estimate the association between NAC and survival in patients with ESCC. We found that patients in the NAC group did not have a significantly improved 5-year OS (HR = 0.81, 95% CI = 0.65–1.00, *P* = 0.053), with significant heterogeneity (*I*^2^ = 58.2%, *P* = 0.035). However, the *P* value was close to 0.05 and the difference was significant at *α* = 0.1. If we discarded RTOG Trial 8911,^7^ there was no heterogeneity in the analysis with a *P* < 0.001 and an *I*^2^ value of 0%. And the 5-year survival for NAC was HR = 0.73, 95% CI = 0.63 to 0.86, *P* < 0.001. From Table 2, we could find RTOG Trial 8911 had 123 multicenters with recruitment for 5 years and 4 months. Three cycles of NAC, higher dose of chemotherapy might be the other 2 detrimental problems. These problems may contribute to the heterogeneity. The cisplatin and 5-fluorouracil (CF) was used for the NAC protocol in all 6 trials. A multicenter phase II feasibility study that examined NAC with docetaxel, cisplatin, and 5-fluorouracil (DCF) for the treatment of ESCC was completed in Japan.^27^ Based on Response Evaluation Criteria in Solid Tumors (RECIST), the overall response rate after the completion of DCF was achieved in 64.3% of the patients. A pathologically complete response was achieved in 17% of the patients.^27^ The updated chemotherapy regimens should be evaluated.

In light of the results of 6 trials and the retrospective analysis of our institute, we are going to begin a multicenter RCT in China to compare NAC cisplatin and paclitaxel (TP) with surgery alone for ESCC (Clinical Trial Registration Number: NCT02395705). It will include level IIIA institutes in different provinces from south to north China and we plan to enroll 528 patients in 2 years. We will use past trials to gain insight for the design of this trial. To this end, we will define the details of the surgical procedures and the range of lymphadenectomies, shorten the interval between NAC treatment and surgery, and adopt the chemotherapy regimens TP. Thus, we hope to help establish a combined therapeutic strategy for ESCC in China.
